# Urea–adipic acid (2/1)

**DOI:** 10.1107/S1600536811015273

**Published:** 2011-05-07

**Authors:** Hai-Sheng Chang, Jian-Li Lin

**Affiliations:** a Center of Applied Solid State Chemistry Research, Ningbo University, Ningbo, Zhejiang 315211, People’s Republic of China

## Abstract

The asymmetric unit of the title co-crystal, 2CH_4_N_2_O·C_6_H_10_O_4_, contains two urea mol­ecules and two half-mol­ecules of adipic acid; the latter are completed by crystallographic inversion symmetry. The crystal packing is stabilized by O—H⋯O and N—H⋯O hydrogen bonds, generating a chain along [110]. Additional weak inter-chain O—H⋯O and N—H⋯O inter­molecular inter­actions lead to the formation of a three-dimensional network.

## Related literature

For urea inclusion compounds, see: Videnova-Adrabińska (1996*a* ▶); Harris & Thomas (1990 ▶); Yeo *et al.* (1997 ▶). For urea–dicarb­oxy­lic acid co-crystal engineering with predesigned crystal building blocks, see: Videnova-Adrabińska (1996*b* ▶). For a urea-dicarb­oxy­lic acid co-crystal with a phase diagram, see: Chadwick *et al.* (2009 ▶).
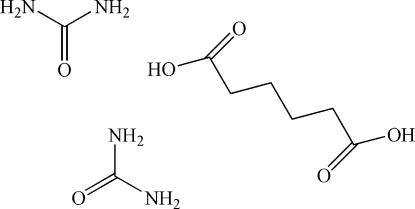


## Experimental

### 

#### Crystal data

2CH_4_N_2_O·C_6_H_10_O_4_
*M* *_r_* = 266.26
Triclinic, 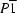

*a* = 7.2484 (14) Å
*b* = 7.6965 (15) Å
*c* = 11.964 (2) Å
α = 101.81 (3)°
β = 92.55 (3)°
γ = 91.92 (3)°
*V* = 652.0 (2) Å^3^
*Z* = 2
Mo *K*α radiation
μ = 0.12 mm^−1^
*T* = 293 K
0.35 × 0.26 × 0.18 mm

#### Data collection

Rigaku R-AXIS RAPID diffractometer
Absorption correction: multi-scan (*ABSCOR*; Higashi, 1995 ▶) *T*_min_ = 0.965, *T*_max_ = 0.980
6479 measured reflections
2949 independent reflections
1457 reflections with *I* > 2σ(*I*)
*R*_int_ = 0.048

#### Refinement

*R*[*F* ^2^ > 2σ(*F* ^2^)] = 0.049
*wR*(*F* ^2^) = 0.186
*S* = 1.11
2949 reflections
164 parameters
H-atom parameters constrained
Δρ_max_ = 0.37 e Å^−3^
Δρ_min_ = −0.35 e Å^−3^

### 

Data collection: *RAPID-AUTO* (Rigaku, 1998 ▶); cell refinement: *RAPID-AUTO*; data reduction: *CrystalStructure* (Rigaku/MSC, 2004 ▶); program(s) used to solve structure: *SHELXS97* (Sheldrick, 2008 ▶); program(s) used to refine structure: *SHELXL97* (Sheldrick, 2008 ▶); molecular graphics: *SHELXTL* (Sheldrick, 2008 ▶); software used to prepare material for publication: *SHELXTL*.

## Supplementary Material

Crystal structure: contains datablocks global, I. DOI: 10.1107/S1600536811015273/jj2086sup1.cif

Structure factors: contains datablocks I. DOI: 10.1107/S1600536811015273/jj2086Isup2.hkl

Additional supplementary materials:  crystallographic information; 3D view; checkCIF report

## Figures and Tables

**Table 1:** Hydrogen-bond geometry (Å, °)

*D*—H⋯*A*	*D*—H	H⋯*A*	*D*⋯*A*	*D*—H⋯*A*
O2—H2*E*⋯O6	0.84	1.78	2.611 (3)	173
O4—H4*C*⋯O5	0.84	1.77	2.588 (3)	167
N1—H1*A*⋯O5^i^	0.86	2.09	2.942 (3)	172
N1—H1*B*⋯O2^ii^	0.86	2.42	3.203 (3)	151
N2—H2*A*⋯O3	0.86	2.20	3.031 (4)	164
N2—H2*B*⋯O2^ii^	0.86	2.38	3.171 (4)	154
N3—H3*A*⋯O1	0.86	2.08	2.912 (4)	163
N3—H3*B*⋯O4	0.86	2.34	3.049 (3)	140
N4—H4*A*⋯O6^iii^	0.86	2.11	2.956 (3)	170
N4—H4*B*⋯O3^iv^	0.86	2.26	3.055 (3)	155

Symmetry codes: (i) 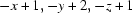; (ii) 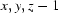; (iii) 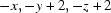; (iv) 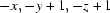.

**Fig. 1.**
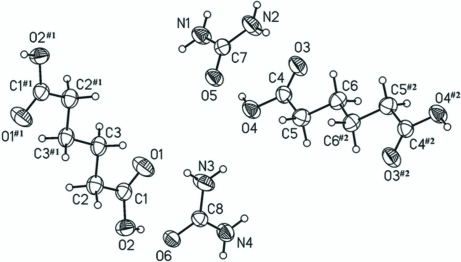
Molecular structure of the title co-crystal. Displacement ellipsoids are shown at the 45% probability level.(#1 = -x + 2, -y + 1, -z + 2; #2 = -x, -y, -z + 1)

**Fig. 2.**
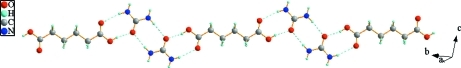
One-dimensional chain of the title co-crystal viewed along the b axis. O–H···O and N–H···O hydrogen bonds are shown as dashed lines.

**Fig. 3.**
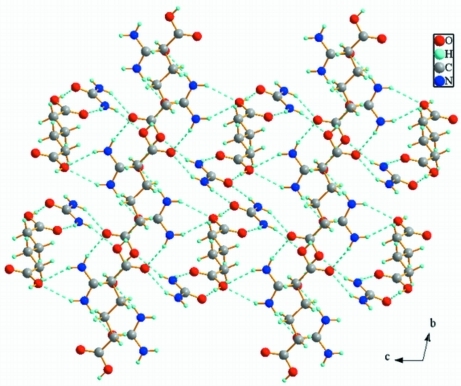
Packing diagram of the title co-crystal viewed down the a axis. O–H···O and N–H···O hydrogen bonds are shown as dashed lines.

## supplementary crystallographic information

### Comment

There is considerable interest in the structural and dynamic properties of urea inclusion compounds. In these solids (Videnova-Adrabińska, 1996*a*), the urea molecules form an extensively hydrogen-bonded host structure (Harris *et al.*, 1990), containing linear, parallel tunnels with guest molecules packed densely along these tunnels (Yeo *et al.*, 1997). This crystal structure study is part of a broader program of urea-dicarboxylic acid cocrystal engineering with predesigned crystal building blocks (Videnova-Adrabińska 1996*b*). The phase diagram of a related urea-dicarboxylic co-crystal has also been reported (Chadwick *et al.*, 2009). In this contribution, we report the title compound with Urea–adipic acid cocrystals (2:1) which form an extensively hydrogen-bonded three-dimensional supramolecular architecture.

The asymmetric unit contains two urea molecules and two half-adipic acid molecules, with the complete adipic acid molecule generated *via* crystallographic inversion symmetry (Fig. 1). The carboxylic groups of adipic acid connect with the corresponding urea molecules and inter-urea through O4–H4C···O5, N2–H2A···O3 and N1–H1A···O5^iii^ (Table. 1) hydrogen bonds generating a one-dimensional chain along [110] (Fig. 2). Nearby, mutually perpendicular chairs are connected in a similar fashion forming a chain with O2–H2E···O6, N3–H3A···O1 and N4–H4A···O6^v^ hydrogen bond interactions. Additional weak inter-chain O–H···O and N–H···O intermolecular interactions (Table. 1) support an extensive three-dimensional network, which consolidates the crystal packing (Fig. 3).

### Experimental

Adipic acid (0.0371 g, 0.25 mmol) and urea (0.0360 g, 0.60 mmol) were dissolved in 15 ml water (pH = 3.11) under stirring. After slow evaporation of the solution for one week at 50°C, colorless block crystals were formed.

### Refinement

H atoms bonded to C atoms were placed in their geometrically calculated positions and refined using the riding model, with C–H distances 0.97 Å, N–H distances 0.86Å and *U*_iso_(H) = 1.2 *U*_eq_(C, N). H atoms attached to O atoms were found in a difference Fourier map and then refined using the riding model, with O–H distances fixed as initially found and with *U*_iso_(H) values set at 1.2 *U*_eq_(O).

### Crystal data

**Table:**

2CH_4_N_2_O·C_6_H_10_O_4_	*Z* = 2
*M**_r_* = 266.26	*F*(000) = 284
Triclinic, *P*1	*D*_x_ = 1.356 Mg m^−^^3^
Hall symbol: -P 1	Mo *K*α radiation, λ = 0.71073 Å
*a* = 7.2484 (14) Å	Cell parameters from 3579 reflections
*b* = 7.6965 (15) Å	θ = 3.2–27.5°
*c* = 11.964 (2) Å	µ = 0.12 mm^−^^1^
α = 101.81 (3)°	*T* = 293 K
β = 92.55 (3)°	Block, colorless
γ = 91.92 (3)°	0.35 × 0.26 × 0.18 mm
*V* = 652.0 (2) Å^3^	

### Data collection

**Table:**

Rigaku R-AXIS RAPID diffractometer	2949 independent reflections
Radiation source: fine-focus sealed tube	1457 reflections with *I* > 2σ(*I*)
graphite	*R*_int_ = 0.048
ω scans	θ_max_ = 27.5°, θ_min_ = 3.2°
Absorption correction: multi-scan (*ABSCOR*; Higashi, 1995)	*h* = −9→8
*T*_min_ = 0.965, *T*_max_ = 0.980	*k* = −9→9
6479 measured reflections	*l* = −15→15

### Refinement

**Table:**

Refinement on *F*^2^	Secondary atom site location: difference Fourier map
Least-squares matrix: full	Hydrogen site location: inferred from neighbouring sites
*R*[*F*^2^ > 2σ(*F*^2^)] = 0.049	H-atom parameters constrained
*wR*(*F*^2^) = 0.186	*w* = 1/[σ^2^(*F*_o_^2^) + (0.0647*P*)^2^ + 0.4241*P*] where *P* = (*F*_o_^2^ + 2*F*_c_^2^)/3
*S* = 1.11	(Δ/σ)_max_ < 0.001
2949 reflections	Δρ_max_ = 0.37 e Å^−^^3^
164 parameters	Δρ_min_ = −0.35 e Å^−^^3^
0 restraints	Extinction correction: *SHELXL97* (Sheldrick, 2008), Fc^*^=kFc[1+0.001xFc^2^λ^3^/sin(2θ)]^-1/4^
Primary atom site location: structure-invariant direct methods	Extinction coefficient: 0.072 (9)

### Special details

**Table:**

Geometry. All e.s.d.'s (except the e.s.d. in the dihedral angle between two l.s. planes) are estimated using the full covariance matrix. The cell e.s.d.'s are taken into account individually in the estimation of e.s.d.'s in distances, angles and torsion angles; correlations between e.s.d.'s in cell parameters are only used when they are defined by crystal symmetry. An approximate (isotropic) treatment of cell e.s.d.'s is used for estimating e.s.d.'s involving l.s. planes.
Refinement. Refinement of *F*^2^ against ALL reflections. The weighted *R*-factor *wR* and goodness of fit *S* are based on *F*^2^, conventional *R*-factors *R* are based on *F*, with *F* set to zero for negative *F*^2^. The threshold expression of *F*^2^ > σ(*F*^2^) is used only for calculating *R*-factors(gt) *etc*. and is not relevant to the choice of reflections for refinement. *R*-factors based on *F*^2^ are statistically about twice as large as those based on *F*, and *R*- factors based on ALL data will be even larger.

### Fractional atomic coordinates and isotropic or equivalent isotropic displacement parameters (Å^2^)

**Table:**

	*x*	*y*	*z*	*U*_iso_*/*U*_eq_	
O1	0.5934 (4)	0.6694 (4)	0.8940 (2)	0.0678 (8)	
O2	0.5075 (3)	0.7886 (3)	1.06743 (19)	0.0478 (6)	
H2E	0.4187	0.8216	1.0308	0.072*	
C1	0.6238 (4)	0.7000 (4)	0.9963 (3)	0.0408 (7)	
C2	0.7946 (4)	0.6471 (4)	1.0527 (3)	0.0443 (8)	
H2C	0.7600	0.5941	1.1160	0.053*	
H2D	0.8716	0.7530	1.0838	0.053*	
C3	0.9073 (4)	0.5167 (4)	0.9733 (3)	0.0435 (8)	
H3C	0.8368	0.4046	0.9506	0.052*	
H3D	0.9276	0.5622	0.9048	0.052*	
O3	0.1850 (4)	0.3529 (3)	0.37617 (19)	0.0552 (7)	
O4	0.2542 (3)	0.5187 (3)	0.54892 (18)	0.0500 (6)	
H4C	0.2980	0.5916	0.5130	0.075*	
C4	0.1849 (4)	0.3734 (4)	0.4793 (3)	0.0399 (7)	
C5	0.1037 (5)	0.2411 (4)	0.5417 (3)	0.0438 (8)	
H5A	0.0037	0.2953	0.5857	0.053*	
H5B	0.1977	0.2146	0.5952	0.053*	
C6	0.0300 (4)	0.0679 (4)	0.4655 (3)	0.0422 (8)	
H6A	0.1253	0.0190	0.4153	0.051*	
H6B	−0.0745	0.0914	0.4182	0.051*	
O5	0.3998 (3)	0.7766 (3)	0.46884 (18)	0.0477 (6)	
C7	0.4225 (4)	0.7824 (4)	0.3659 (3)	0.0404 (7)	
N1	0.4904 (4)	0.9306 (3)	0.3376 (2)	0.0522 (8)	
H1A	0.5191	1.0232	0.3900	0.063*	
H1B	0.5054	0.9332	0.2671	0.063*	
N2	0.3789 (5)	0.6436 (4)	0.2825 (2)	0.0600 (9)	
H2A	0.3341	0.5469	0.2982	0.072*	
H2B	0.3953	0.6499	0.2127	0.072*	
O6	0.2201 (3)	0.9024 (3)	0.96911 (17)	0.0419 (6)	
C8	0.1495 (4)	0.8482 (4)	0.8697 (3)	0.0370 (7)	
N3	0.2432 (4)	0.7456 (4)	0.7892 (2)	0.0564 (8)	
H3A	0.3532	0.7161	0.8053	0.068*	
H3B	0.1935	0.7093	0.7215	0.068*	
N4	−0.0194 (4)	0.8939 (3)	0.8407 (2)	0.0466 (7)	
H4A	−0.0815	0.9615	0.8904	0.056*	
H4B	−0.0660	0.8558	0.7723	0.056*	

### Atomic displacement parameters (Å^2^)

**Table:**

	*U*^11^	*U*^22^	*U*^33^	*U*^12^	*U*^13^	*U*^23^
O1	0.0634 (17)	0.0839 (18)	0.0446 (16)	0.0303 (14)	−0.0110 (13)	−0.0150 (13)
O2	0.0428 (13)	0.0601 (14)	0.0419 (13)	0.0105 (11)	0.0071 (10)	0.0118 (11)
C1	0.0391 (18)	0.0333 (15)	0.048 (2)	0.0012 (13)	0.0018 (15)	0.0027 (14)
C2	0.0412 (18)	0.0391 (16)	0.052 (2)	0.0007 (14)	−0.0008 (15)	0.0094 (15)
C3	0.0404 (18)	0.0329 (15)	0.055 (2)	−0.0023 (13)	−0.0013 (15)	0.0056 (14)
O3	0.0784 (18)	0.0461 (13)	0.0371 (14)	−0.0182 (12)	0.0011 (12)	0.0028 (10)
O4	0.0681 (16)	0.0402 (12)	0.0383 (13)	−0.0146 (11)	0.0061 (11)	0.0023 (10)
C4	0.0424 (18)	0.0352 (15)	0.0406 (19)	−0.0028 (13)	0.0007 (14)	0.0056 (13)
C5	0.0489 (19)	0.0397 (16)	0.0441 (19)	−0.0006 (14)	0.0061 (15)	0.0117 (14)
C6	0.0426 (18)	0.0365 (15)	0.0493 (19)	−0.0005 (13)	0.0053 (15)	0.0124 (14)
O5	0.0645 (16)	0.0396 (12)	0.0363 (13)	−0.0118 (11)	0.0069 (11)	0.0023 (9)
C7	0.0412 (18)	0.0370 (16)	0.0407 (18)	−0.0024 (13)	0.0043 (14)	0.0028 (13)
N1	0.074 (2)	0.0440 (15)	0.0369 (15)	−0.0141 (14)	0.0108 (14)	0.0057 (12)
N2	0.090 (2)	0.0472 (16)	0.0367 (16)	−0.0175 (16)	0.0021 (15)	−0.0016 (13)
O6	0.0444 (13)	0.0450 (12)	0.0329 (12)	0.0059 (10)	−0.0015 (10)	0.0004 (9)
C8	0.0424 (18)	0.0330 (15)	0.0346 (17)	0.0004 (13)	0.0029 (14)	0.0049 (13)
N3	0.063 (2)	0.0648 (18)	0.0359 (16)	0.0191 (16)	0.0028 (14)	−0.0042 (13)
N4	0.0459 (17)	0.0509 (15)	0.0399 (15)	0.0055 (13)	−0.0052 (12)	0.0032 (12)

### Geometric parameters (Å, °)

**Table:**

O1—C1	1.207 (4)	C6—C6^ii^	1.522 (6)
O2—C1	1.329 (4)	C6—H6A	0.9700
O2—H2E	0.8383	C6—H6B	0.9700
C1—C2	1.492 (4)	O5—C7	1.259 (4)
C2—C3	1.520 (4)	C7—N2	1.323 (4)
C2—H2C	0.9700	C7—N1	1.339 (4)
C2—H2D	0.9700	N1—H1A	0.8600
C3—C3^i^	1.515 (6)	N1—H1B	0.8600
C3—H3C	0.9700	N2—H2A	0.8600
C3—H3D	0.9700	N2—H2B	0.8600
O3—C4	1.212 (4)	O6—C8	1.257 (3)
O4—C4	1.319 (4)	C8—N4	1.335 (4)
O4—H4C	0.8357	C8—N3	1.340 (4)
C4—C5	1.500 (4)	N3—H3A	0.8600
C5—C6	1.518 (4)	N3—H3B	0.8600
C5—H5A	0.9700	N4—H4A	0.8600
C5—H5B	0.9700	N4—H4B	0.8600
			
C1—O2—H2E	110.5	H5A—C5—H5B	107.5
O1—C1—O2	121.8 (3)	C5—C6—C6^ii^	112.1 (3)
O1—C1—C2	123.4 (3)	C5—C6—H6A	109.2
O2—C1—C2	114.8 (3)	C6^ii^—C6—H6A	109.2
C1—C2—C3	113.9 (3)	C5—C6—H6B	109.2
C1—C2—H2C	108.8	C6^ii^—C6—H6B	109.2
C3—C2—H2C	108.8	H6A—C6—H6B	107.9
C1—C2—H2D	108.8	O5—C7—N2	121.3 (3)
C3—C2—H2D	108.8	O5—C7—N1	120.7 (3)
H2C—C2—H2D	107.7	N2—C7—N1	118.0 (3)
C3^i^—C3—C2	113.4 (3)	C7—N1—H1A	120.0
C3^i^—C3—H3C	108.9	C7—N1—H1B	120.0
C2—C3—H3C	108.9	H1A—N1—H1B	120.0
C3^i^—C3—H3D	108.9	C7—N2—H2A	120.0
C2—C3—H3D	108.9	C7—N2—H2B	120.0
H3C—C3—H3D	107.7	H2A—N2—H2B	120.0
C4—O4—H4C	111.7	O6—C8—N4	121.0 (3)
O3—C4—O4	122.7 (3)	O6—C8—N3	120.8 (3)
O3—C4—C5	124.5 (3)	N4—C8—N3	118.1 (3)
O4—C4—C5	112.8 (3)	C8—N3—H3A	120.0
C4—C5—C6	114.8 (3)	C8—N3—H3B	120.0
C4—C5—H5A	108.6	H3A—N3—H3B	120.0
C6—C5—H5A	108.6	C8—N4—H4A	120.0
C4—C5—H5B	108.6	C8—N4—H4B	120.0
C6—C5—H5B	108.6	H4A—N4—H4B	120.0

Symmetry codes: (i) −*x*+2, −*y*+1, −*z*+2; (ii) −*x*, −*y*, −*z*+1.

### Hydrogen-bond geometry (Å, °)

**Table:**

*D*—H···*A*	*D*—H	H···*A*	*D*···*A*	*D*—H···*A*
O2—H2E···O6	0.84	1.78	2.611 (3)	173
O4—H4C···O5	0.84	1.77	2.588 (3)	167
N1—H1A···O5^iii^	0.86	2.09	2.942 (3)	172
N1—H1B···O2^iv^	0.86	2.42	3.203 (3)	151
N2—H2A···O3	0.86	2.20	3.031 (4)	164
N2—H2B···O2^iv^	0.86	2.38	3.171 (4)	154
N3—H3A···O1	0.86	2.08	2.912 (4)	163
N3—H3B···O4	0.86	2.34	3.049 (3)	140
N4—H4A···O6^v^	0.86	2.11	2.956 (3)	170
N4—H4B···O3^vi^	0.86	2.26	3.055 (3)	155

Symmetry codes:  (iii) −*x*+1, −*y*+2, −*z*+1; (iv) *x*, *y*, *z*−1; (v) −*x*, −*y*+2, −*z*+2; (vi) −*x*, −*y*+1, −*z*+1.
